# Dynamics of phosphorus and bacterial *phoX* genes during the decomposition of *Microcystis* blooms in a mesocosm

**DOI:** 10.1371/journal.pone.0195205

**Published:** 2018-05-03

**Authors:** Jiangyu Dai, Dan Chen, Shiqiang Wu, Xiufeng Wu, Guang Gao, Xiangming Tang, Keqiang Shao, Xueyan Lv, Wanyun Xue, Qianqian Yang, Senlin Zhu

**Affiliations:** 1 State Key Laboratory of Hydrology-Water Resources and Hydraulic Engineering, Nanjing Hydraulic Research Institute, Nanjing, P. R. China; 2 Nanjing Guohuan Science and Technology Co., Ltd of Nanjing Institute of Environmental Sciences, MEP, Nanjing, P.R. China; 3 Nanjing Institute of Limnology and Geography, Chinese Academy of Sciences, Nanjing, P. R. China; 4 Jiangsu Environmental Monitoring Center, Nanjing, P. R. China; CEA-Saclay, FRANCE

## Abstract

Cyanobacterial blooms are a worldwide environmental problem and frequently occur in eutrophic lakes. Organophosphorus mineralization regulated by microbial alkaline phosphatase provides available nutrients for bloom regeneration. To uncover the dynamics of bacterial alkaline phosphatase activity and microbial backgrounds in relation to organophosphorus mineralization during the decomposition process of cyanobacterial blooms, the response of alkaline phosphatase PhoX-producing bacteria were explored using a 23-day mesocosm experiment with three varying densities of *Microcystis* biomass from eutrophic Lake Taihu. Our study found large amounts of soluble reactive phosphorus and dissolved organophosphorus were released into the lake water during the decomposition process. Bacterial alkaline phosphatase activity showed the peak values during days 5~7 in groups with different chlorophyll-a densities, and then all decreased dramatically to their initial experimental levels during the last stage of decomposition. Bacterial *phoX* abundances in the three experimental groups increased significantly along with the decomposition process, positively related to the dissolved organic carbon and organophosphorus released by the *Microcystis* blooms. The genotypes similar to the *phoX* genes of *Alphaproteobacteria* were dominant in all groups, whereas the genotypes most similar to the *phoX* genes of *Betaproteobacteria* and *Cyanobacteria* were also abundant in the low density (~15 μg L^-1^ chlorophyll-*a*) group. At the end of the decomposition process, the number of genotypes most similar to the *phoX* of *Betaproteobacteria* and *Cyanobacteria* increased in the medium (~150 μg L^-1^ chlorophyll-*a*) and high (~1500 μg L^-1^ chlorophyll-*a*) density groups. The released organophosphorus and increased bacterial *phoX* abundance after decomposition of *Microcystis* aggregates could potentially provide sufficient nutrients and biological conditions for algal proliferation and are probably related to the regeneration of *Microcystis* blooms in eutrophic lakes.

## Introduction

Cyanobacterial blooms are a major ecological problem in many eutrophic lakes worldwide. The accumulation and decomposition of cyanobacterial blooms often result in deteriorating lake water quality and thereby influence the survival and reproduction of aquatic organisms [1–4]. Bacteria is an important component of the microbial food web in lakes and therefore plays a significant role in the cycle of biological matter [5–6]. A large amount of released organic matter from the decomposed cyanobacterial blooms can be transformed by bacteria into inorganic nutrients, which can be directly utilized by autotrophic organisms such as *Microcystis* sp. [7]. In turn, bacterial community composition can also be influenced by the decomposition of cyanobacterial blooms [7–11].

Phosphorus, one of the basic nutrient elements released in the decomposition of cyanobacterial blooms, is an important factor limiting the growth of bacteria and outbreaks of cyanobacterial blooms [12–13]. Previous studies found that a large amount of dissolved enzymatically hydrolysable phosphorus (EHP), such as phosphoric monoesters and phosphodiesters that can be hydrolysed by extracellular alkaline phosphatases (APases)-producing bacteria, were released in the decomposition of cyanobacterial blooms [14–15]. As the critical substrates for bacteria, EHP with different contents could promote or inhibit bacterial growth and APases activity [16] and thus alter bacterial communities during the decomposition of cyanobacterial blooms. Existing studies, however, focus on the changes in bacterial community structure and composition during the accumulation and decomposition of cyanobacterial blooms [7–8, 11, 14–15]. Few have reported on the dynamics of APases-producing bacteria during this process.

Microcystins are always released during the accumulation and decomposition of *Microcystis* blooms [17], posing a significant hazard to lake ecosystem health and water quality. Studying the formation, outbreak, and extinction of *Microcystis* blooms is therefore essential to its control. Previous study has demonstrated that the frequent outbreak of *Microcystis* blooms is directly related to the regeneration of bioavailable phosphorus in lake water [13], whereas the decomposition of *Microcystis* blooms could produce sufficient bioavailable phosphorus such as EHP [18]. To utilize the released EHP in lake water, cyanobacteria need active extracellular APases, of which bacterial extracellular APases are indispensable. However, the dynamics of bacterial APases and its producing bacteria during the decomposition of *Microcystis* blooms remain unknown.

As an important contributor to extracellular bacterial APases, APases PhoX and its encoding genes (*phoX*) have become the focus of research into microbial phosphorus limitation in natural waters [19–23]. Previous studies have demonstrated that bacterial *phoX* were abundant and diverse in some eutrophic lakes [24–25]. Given the important role of PhoX in extracellular bacterial APases, the PhoX-encoding genes have been demonstrated to be available biomarkers [20, 23–26], revealing the APase-producing bacterial information during the organophosphate mineralization process.

Lake Taihu, a large, eutrophic freshwater lake in southeast China, is threatened by frequent and noxious *Microcystis* blooms every summer and autumn [3, 27]. To uncover the dynamics of bacterial extracellular APases and APase PhoX-producing bacteria during the decomposition process of *Microcystis* blooms, an *in situ* mesocosm experiment modelling the decomposition of *Microcystis* blooms with different concentrations of chlorophyll-*a* (Chl-*a*) were studied in the north Meiliang Bay of Lake Taihu. This study will be beneficial for the microbial driving mechanism of organophosphate mineralization during the decomposition of *Microcystis* blooms in eutrophic lakes.

## Materials and methods

### Experimental settings

**Fig 1:**
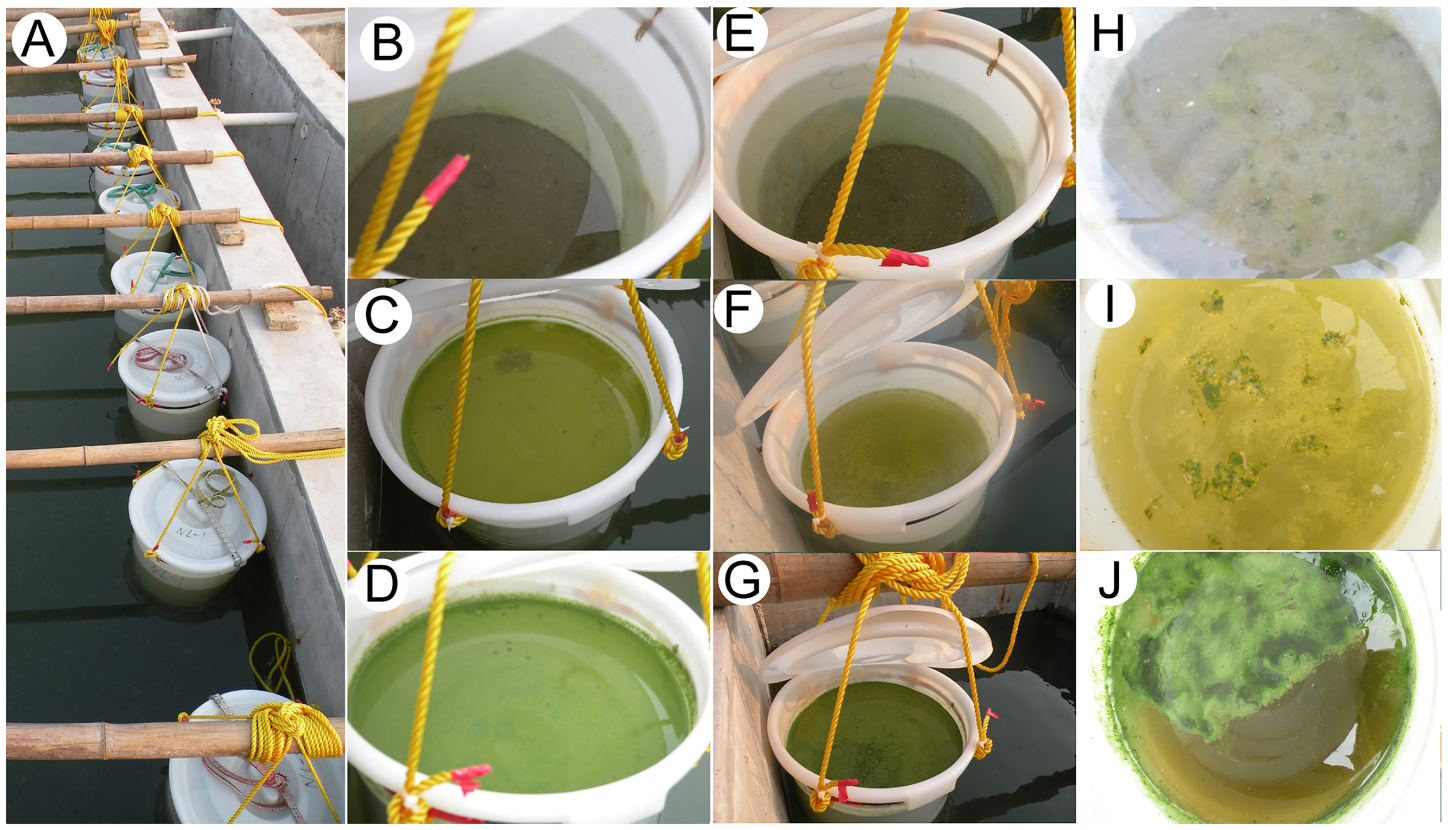
The in situ mesocosm experiment modelling the decomposition process of *Microcystis* blooms with different densities of chlorophyll-*a* concentration.

The Taihu Laboratory for Lake Ecosystem Research (TLLER) is a public research base located beside Lake Taihu for water sciences and technologies about shallow eutrophic Lakes. To maximize simulated field water temperature and sunlight conditions, this mesocosm experiment was conducted in a large artificial flume on the lakeshore in TLLER with the permission of this research base. Before the experiment, a large amount of water collected from the eutrophic Meiliang Bay of Lake Taihu was poured into the flume to create the lake aquatic environment (Fig 1A). Meiliang Bay is a representative hypereutrophic, algae-dominated region in the northern part of the lake [28] that experiences intensive *Microcystis* blooms every summer and autumn [3]. Mixed surface sediments (≤10 cm of sediment depth) were gathered with a Peterson grab sampler (1/40 m^2^ of sampling area) from Meiliang Bay two days before the experiment and placed at the bottom of nine euphotic polyethylene barrels (40 L volume for each) to a thickness of about 5 cm each.

Cyanobacterial aggregates were collected from Meiliang Bay one day before the experiment. Microscopic examination determined that *Microcystis* sp. constituted up to about 98% of the total algal abundance; thus, the cyanobacterial blooms are referred to as *Microcystis* blooms. The fresh water samples were first filtered using nylon gauze with a pore diameter of 150 μm to eliminate apparent *Microcystis* populations. To remove as many impurities and dissolved nutrients as possible, the fresh *Microcystis* aggregates were first filtered using nylon gauze with a 150 μm pore diameter. The collected scum was placed in a clean plastic bucket to which about 10 L distilled water was added mixed lightly. The mixed scums were then refiltered to remove the water. This washing process was repeated for three to four times.

According to the monitoring statistics of Chl-*a* concentrations in Lake Taihu [28], Chl-*a* concentration is maintained at 5–25 μg L^-1^, even when low density blooms of *Microcystis* occur in Lake Taihu during summer. The Chl-*a* concentration of the *Microcystis* blooms in Lake Taihu is about 100–1500 μg L^-1^. In the experiment, the fresh *Microcystis* aggregates were poured into the barrels to establish a gradient of Chl-*a* concentrations: ~15, ~150, and ~1500 μg L^-1^, represented as groups N, L, and H, respectively (Fig 1B, 1C and 1D, respectively). In each group, three replicates were established, and each barrel contained a 30 L water sample.

Based on the decomposition of *Microcystis* blooms, the experiment was performed from 23 August to 14 September 2011. The experimental systems in groups N, L, and H were captured in images during the early (Fig 1B, 1E and 1H), middle (Fig 1C, 1F and 1I), and last stages (Fig 1D, 1G and 1J), respectively, of the experiment.

### Sample collection

On the first day of the experiment (23 August 2011), about 10 g of surface sediments were collected from each barrel before adding fresh *Microcystis* aggregates. These sediment samples were then placed in sterile centrifuge tubes and stored at -20 °C until used to recover the genetic information of bacterial APases PhoX. After adding fresh *Microcystis* aggregates, water samples were then gathered at 7:00 am. A columnar water sampler was slowly placed into the barrels to collect water samples which were then stored in sterile plastic bottles and sent to TLLER. Various water quality parameters were measured during the next 12 hours. After the first sampling, only water column samples were collected in subsequent samplings. During the experiment, all samples were collected at 7:00 am on day 1, 3, 5, 7, 11, 17, and 23.

### Measurements of biotic and abiotic variables

The values of water temperature, pH, dissolved oxygen (DO), and turbidity in each barrel were measured in the field using a YSI 6600 Multi-parameter Water Quality System (YSI, USA). Concentrations of Chl-*a*, total phosphorus (TP), total dissolved phosphorus (TDP), soluble reactive phosphorus (SRP) and dissolved EHP in water samples were determined according to the standard methods used in lake ecosystems [16, 29]. Bacterial APases activity (BAPA) of the water samples was measured using the p-nitrophenyl phosphate (p-NPP) method described in the literature [16, 30]. The dissolved organic carbon (DOC) content of the water samples was automatically quantified by an Elementar TOC Analyzer (Elementar, Shanghai, China) in TLLER. The TP content of the sediment samples was measured using the molybdenum antimony scandium spectrophotometer method [31].

### DNA extraction and polymerase chain reaction amplification

Raw water (100 mL) for each water sample was filtered through a 0.2 μm polycarbonate membrane (Millipore, Ireland). The membrane for each sample was placed into a 2 mL sterile centrifuge tube and stored at -80 °C until DNA extraction using a QIAamp^®^ DNA Mini Kit (QIAGEN, Germany) according to the instructions.

The polymerase chain reaction (PCR) primers (*phoX*-1F/*phoX*-1R; *phoX*-2F/*phoX*-2R; *phoX*-3F/*phoX*-3R; S1 Table) for bacterial *phoX* genes were used according to the previous study [20]. The length of the *phoX* gene fragments obtained were in the range of 600 bp~750 bp. The reaction system for PCR amplification followed the details described in the literature [25]. The templates for PCR positive and negative controls were plasmid *phoX* gene and sterile water, respectively.

PCR amplification was performed in a thermocycler (Applied Biosystems, China) as described in the previous study [25]. After PCR amplification, agarose gel electrophoresis was performed on the amplification products. By using the DL2000 marker as the molecular marker (Takara, China), the size of the product segments was observed through an Omega 10^™^ gel documentation system (Ultra-Lum Inc., USA) to determine whether the amplification was successful.

### Clone library construction and sequencing

This study established the clone libraries based on PCR products of water samples on days 1, 5 (during which bacterial APases activities reached a maximum), and 23. In addition, PCR products of freeze-dried sediments collected on day 1 were mixed to build the clone libraries. The available PCR products of the samples collected on the same day in the same group were mixed. The mixture was purified and recycled using an AxyPrep^®^ DNA Gel Purification Kit (Axygen Company, Hangzhou, China) to generate purified PCR products. The sequences of bacterial *phoX* fragments were determined using an automatic ABI 3730 DNA sequencer (Applied Biosystems, China). About 100 to 120 positive clones of bacterial *phoX* genes were acquired in each clone library.

### Alpha-diversity and phylogenetic analysis

The vector sequences on both ends of all *phoX* gene segments were removed using Bioedit software (version 7.0.9) [32], and the *phoX* segments without vector sequences were then translated into amino acid sequences using the Translate module of the software. Sequences with stop codons were eliminated. According to the quality of the obtained gene fragments, ~600–750 bp lengths of segment sequences that could be successfully translated into amino acid were reserved. The edited sequences were submitted to the online software Bellerphon to remove any chimeral sequences [33]. Valid sequences were then submitted to the GenBank database of National Center for Biotechnology Information (NCBI) to obtain the accession numbers of the gene sequences.

The valid gene sequences obtained were compared using the MUSCLE program [34], and the generated files in phylip format were analysed for nucleic acid distance using the DNAdist program [35]. The similarities in the generated files, as analysed, were compared by the Dotur program to acquire coverage (*C*), Chao 1 richness estimator (*S*_*chao*_), and the Shannon-Wiener index (*H’*) in those clone libraries [36]. Here, the *phoX* sequences with a ≥97% similarity were defined as an operational taxonomic unit (OTU) [24]. Because no similarity cut-off was recognised for bacterial *phoX* OTU determination, a ≥97% similarity was used in the previous [24] and present study as a cut-off.

Based on ≥97% similarity, representative nucleotide sequences of all OTUs were compared for similarity within the NCBI GenBank using BLASTX software (http://www.ncbi.nlm.nih.gov/BLAST/) [24]. The amino acid sequence of the obtained *phoX* gene with highest homology was adopted as the reference sequence for phylogenetic analysis. The evolutionary distance was then calculated using phylogeny analysis software RAxML (version 7.2.8) [37] according to the Jones-Taylor-Thornton model based on a Linux platform. The phylogenetic tree of the amino acid sequence corresponding to the *phoX* gene was established with the maximum likelihood method. The confidence value for topological analysis of the phylogenetic tree was computed by 500-fold random sampling.

Distance analysis was conducted for the PhoX phylogenetic tree constructed with the maximum likelihood method using online software UniFrac (http://unifrac.colorado.edu/) within each clone library [38]. The environmental distance matrix method was applied to perform normalised weighted analysis to obtain the UniFrac distance matrix of the clone library. The cluster plot results of the UniFrac distance matrix analysis were generated using the multivariate statistical analysis software package PRIMER V6 (PRIMER E, Ltd, UK) [39].

### Estimates of bacterial *phoX* gene abundance

From the aforementioned PCR results, the positive *phoX* gene fragments were only observed in the PCR samples amplified using the primers *phoX*-2F/*phoX*-2R [20]. The real-time PCR for quantifying bacterial *phoX* gene abundance was conducted using a Mastercycler^®^ ep realplex (Eppendorf, Shanghai, China) with SYBR^®^ Premix ExTaq Kits (TaKaRa, Dalian, China) and the primers *phoX*-2F/*phoX*-2R.

Each 20 μL reaction contained 0.05 μM of each primer, 10.0 μL of SYBR Premix ExTaq polymerase (TaKaRa, Dalian, China), and 1 μL of template DNA. The thermal profiles were as follows: 94 °C for 30 s, 30 cycles of 94 °C for 15 s, 52 °C for 30 s, and 72 °C for 30 s, followed by a melting curve analysis. The fluorescence intensity was measured at 85 °C for bacterial *phoX*. Standard curves were generated using plasmids extracted from *phoX* clone libraries. Bacterial *phoX* gene standard curves were 10-fold dilutions, with the *phoX* copy numbers ranging from 2.32×10^3^ copies mL^-1^ to 2.32×10^8^ copies mL^-1^. All reactions were run in triplicate. In cases where the *C*_*t*_ value margin exceeded 0.5 among the triplicates per sample, the triplicates were eliminated and a second analysis was performed. Standard curve *R*^2^ values in this assay were >0.98 for all reactions. The efficiencies for bacterial *phoX* real-time PCR ranged from 1.00 to 1.07, with a mean of 1.03.

### Statistical analyses

Variations in the physicochemical parameters and APases activities of water samples among different experimental groups were compared using repeated measures ANOVA test with the Statistical Program for Social Sciences (SPSS) software package 16.0 (IBM, USA), and the plots were drawn using Sigmaplot v12.5 software (Systat Software Inc, USA). Canonical correspondence analysis (CCA) in the software CANOCO 4.53 for Windows [40] was used to investigate the correlation of physicochemical parameters with variations of genotype constitution. Before the analysis, the matrix of genotypes and physicochemical parameters were log_10_ (*x*+1) transformed to ensure they met the requirements for subsequent statistical analysis [41]. The forward selection method in the CANOCO software was used to screen the physicochemical parameters significantly correlated with the genotype constitution. The significance (*P*<0.05) of the calculated results was verified with an unrestricted Monte Carlo permutation [40]. The Pearson correlation and linear regression between the log_10_-transformed bacterial *phoX* gene abundances and water physicochemical parameters were also analysed using the SPSS v16.0 and Sigmaplot v12.5, respectively. Only the significant correlations were plotted with the Sigmaplot v12.5 software.

### Nucleotide sequence accession numbers

The 1050 valid sequences of bacterial *phoX* genes were deposited in the NCBI GenBank database under the accession numbers KC141252-KC141457, KC141459-KC141471, KC141473-KC141528, KC141530-KC141532, KC141534-KC141548, KC141550-KC141643, KC141645-KC141648, KC141650-KC141736, KC141738-KC141832, KC141834-KC141906, KC141909-KC141963, KC299556-KC299905.

## Results

### Physicochemical parameters during the decomposition of *Microcystis* blooms

**Fig 2:**
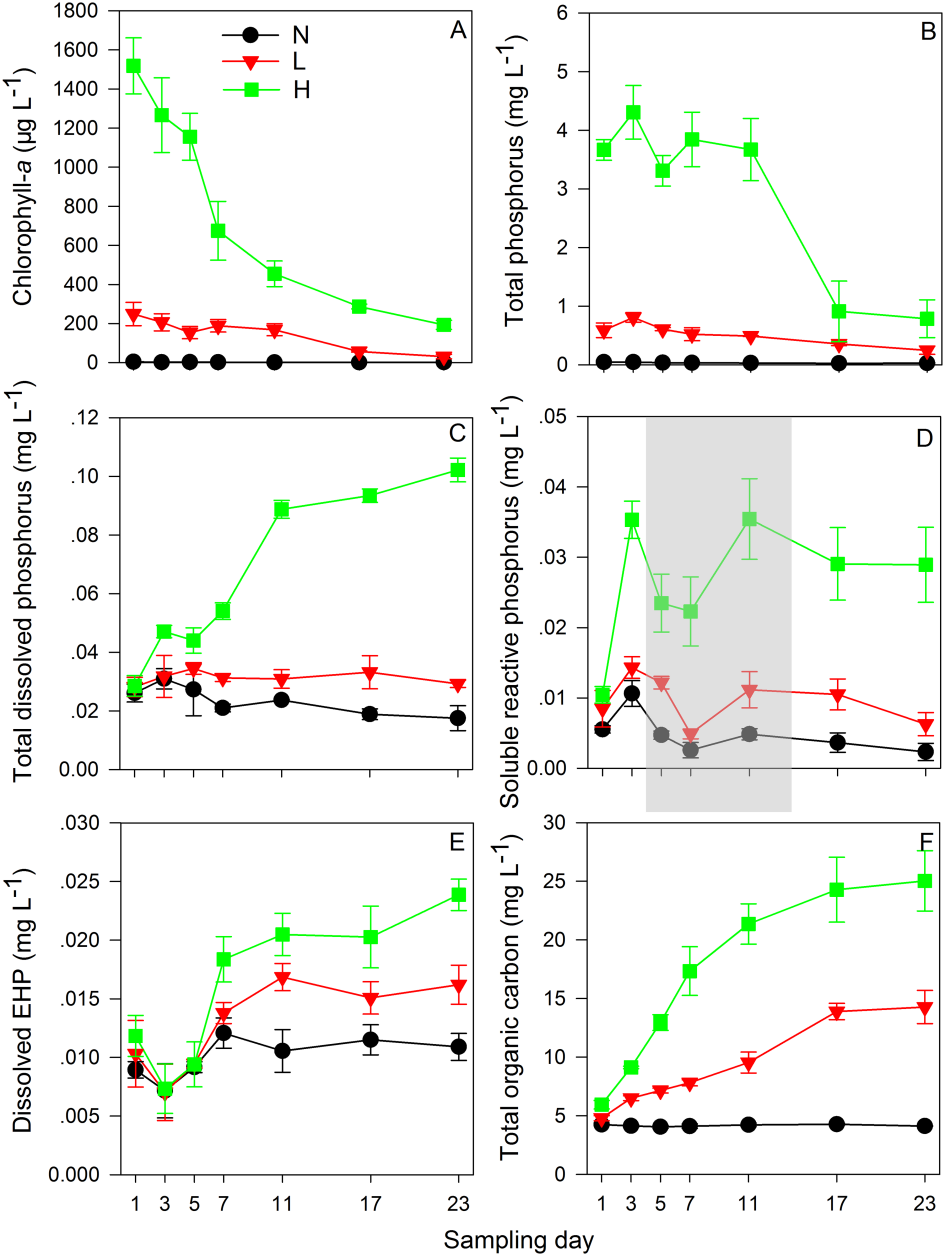
Variations in contents of chlorophyll-a (A), total phosphorus (B), total dissolved phosphorus (C), soluble reactive phosphorus (D), and dissolved enzymatically hydrolysable phosphorus (E) and dissolved organic carbon (D) in water during the simulated decomposition of *Microcystis* blooms. N, L and H in the legend represent the three groups with ~15, ~150 and ~1500μg L^-1^ chlorophyll-*a*, respectively. The grey shadow emphasizes the decrease trend of soluble reactive phosphorus in this experiment.

Water temperature in each group was maintained between 25 and 27 °C during the whole process, except for three days owing to cooler weather (S1A Fig). The average values of the water turbidity in groups L (~150 μg L^-1^ Chl-*a*) and H (~1500 μg L^-1^ Chl-*a*) decreased significantly during the experiment (one-way ANOVA, *P*<0.05; S1B Fig). The average values of water DO contents and pH in three groups all decreased on day 3 and increased on day 5 of the experiment (S1C and S1D Fig). After day 11, both the average values of DO contents and pH in groups L and H decreased. Moreover, significant differences were found in the DO content and pH among the three groups throughout the experiment (repeated measures, *P*<0.05; S1C and S1D Fig). Variations of the Chl-*a* contents in the three experimental groups showed that the Chl-*a* in the L and H groups significantly decreased from day 5 (Fig 2A). By contrast, no significant variation was found in the average content of Chl-*a* in group N throughout the experiment.

The water TP contents in groups L and H significantly decreased during the decomposition of *Microcystis* blooms, whereas the variation in group N was not evident (Fig 2B). On day 23 of this experiment, the enrichment of TP in sediments was evident in the three groups, with the gradient of enrichment following H>L>N (one-way ANOVA, *P*<0.05; S2 Fig). Both the water TDP and SRP contents in group H significantly increased during the experiment, whereas variations in groups N and L were not evident (Fig 2C and 2D). Similar to water temperature variations, DO content and pH in the three groups (S1 Fig) and the water SRP contents (Fig 2D) all decreased on day 3 and 5. The dissolved EHP and DOC contents in groups L and H increased significantly during the experiment (Fig 2E and 2F). During the decomposition of *Microcystis* blooms, differences in phosphorus and DOC contents were evident among the three groups, with the gradient of contents following H>L>N (repeated measures, *P*<0.05; Fig 2).

### Bacterial APases activities

**Fig 3:**
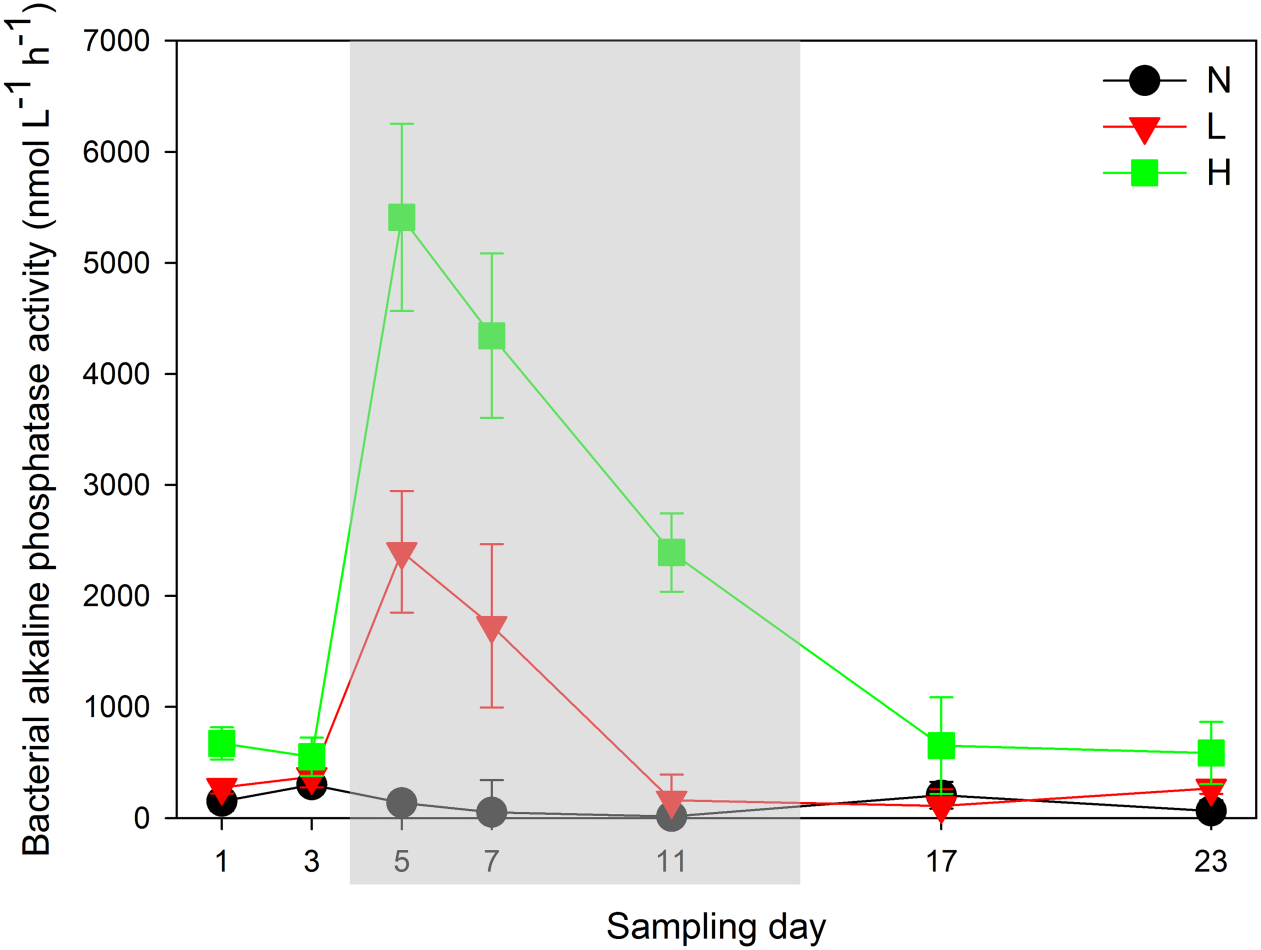
Variations of bacterial alkaline phosphatase activity in different experimental groups. N, L and H in the legend represent the three groups with ~15, ~150 and ~1500μg L^-1^ chlorophyll-*a*, respectively. The grey shadow emphasizes the peak values of bacterial alkaline phosphatase activity in this experiment.

BAPA in groups L and H showed the same trend in variations during the decomposition of *Microcystis* blooms (Fig 3). Both reached peak values on day 5 and then decreased along with *Microcystis* bloom decomposition. By contrast, the variation in group N was not evident. Significant differences in BAPA among the three groups was noted during the experiment, in the decreasing order of groups H, L, and N (repeated measures, *P*<0.05).

### Alpha diversity of bacterial *phoX* genes

During the experiment, bacterial *phoX* genes were found in water samples on day 1, 5, and 23 in the three treatment groups (Table 1). The coverage range of the clone libraries for water samples was 83.1% to 98.0%. The alpha diversity indexes (OTUs, *S*_*Chao*_, and *H’*) of bacterial *phoX* genes in surface sediments were higher than those of water samples in different groups, but the common *phoX* genotypes in both water and sediment samples only accounted for 5.7% of all *phoX* genotypes found in this study. The *S*_*Chao*_ and *H’* diversity indexes in groups L and H on day 5 were lower than those on day 1 and 23, respectively, whereas the variation of diversity in group N was not evident. In this experiment, no obvious difference was found in the average diversity of bacterial *phoX* genes between the L and H groups (one-way ANOVA, *P* > 0.05).

**Table 1:** Clones, operational taxonomic units (OTUs), Chao 1 richness estimator (*S*_*Chao*_), Shannon-Wiener index (*H’*), and coverage (*C*) of bacterial *phoX* gene clone libraries at similarity levels ≥97%.

Library*	Clones	OTUs	*S*_*Chao*_	*H’*	*C* (%)
N-1	112	18	21.0	2.02	94.6
N-5	71	19	23.0	2.13	83.1
N-23	100	16	22.3	2.10	90.0
L-1	119	21	24.5	2.12	96.7
L-5	117	14	15.2	1.43	94.2
L-23	113	18	22.0	2.20	90.3
H-1	108	23	28.5	2.26	97.2
H-5	99	8	9.0	1.16	98.0
H-23	94	18	26.0	2.24	90.4
S	117	30	45.0	2.80	87.2

* N-1, N-5, N-23, L-1, L-5, L-23, H-1, H-5, and H-23 represent the clone libraries constructed from the groups with varying densities of *Microcystis* biomass ranging from 15 to 1500 μg L^-1^ chlorophyll-*a* on day 1, 5, and 23, respectively; S represents the clone library constructed by the sedimentary sample on day 1.

### Phylogenetic analysis of bacterial *phoX* genes

The phylogenetic trees of the protein sequences of *phoX* genotypes in the clone libraries and the reference protein sequences of PhoX in GenBank (S3 Fig) showed that bacterial *phoX* genotypes obtained in this study could be divided into two phylogenetic groups (Cluster I and Cluster II). The *phoX* genotypes in Cluster II were most similar to those of *Alphaproteobacteria*, whereas Cluster I contained the genotypes most similar to the *phoX* genes of *Betaproteobacteria*, *Gammaproteobacteria*, *Epsilonproteobacteria*, and *Cyanobacteria*.

**Fig 4:**
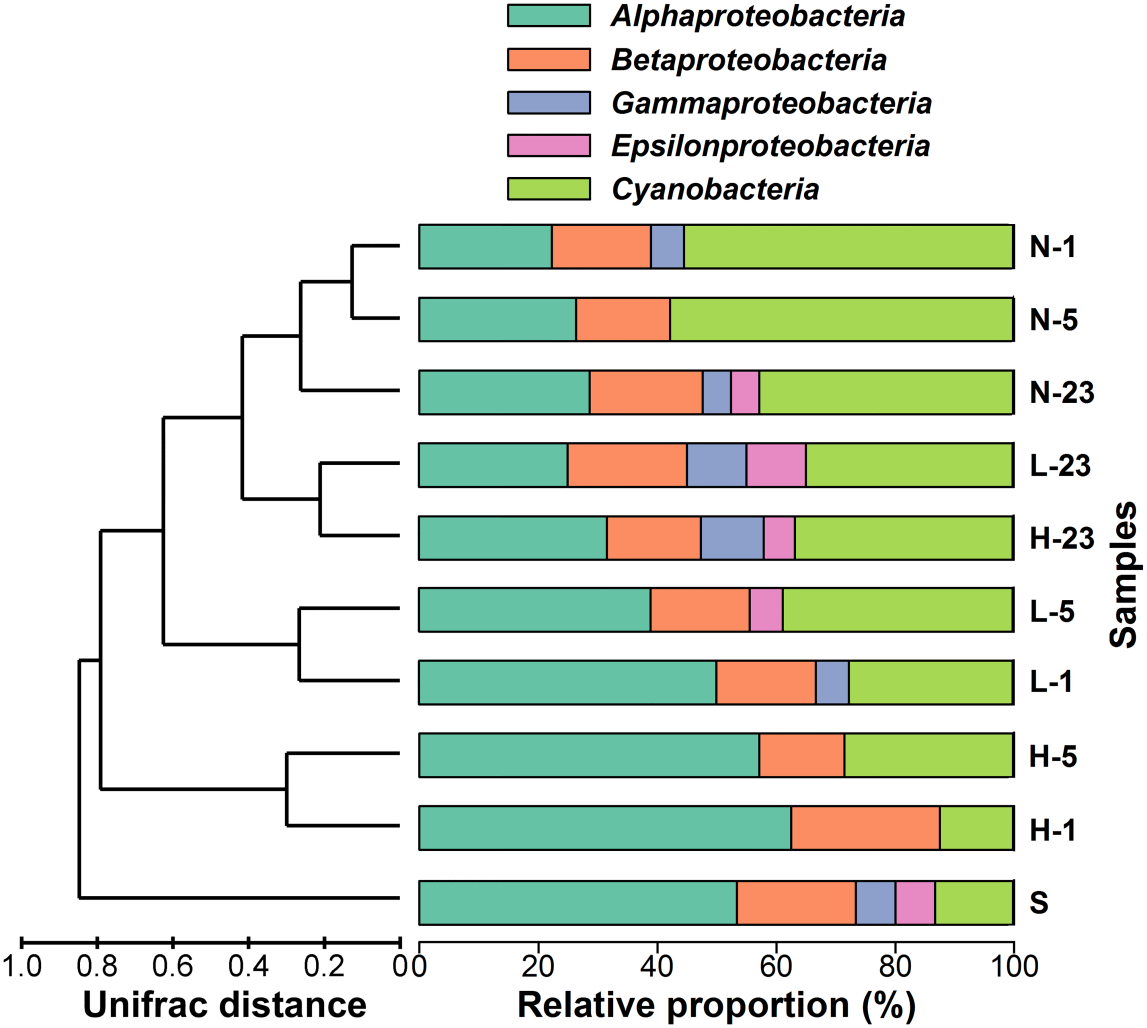
Cluster analysis tree based on the UniFrac distances calculated from the phylogenetic tree of bacterial *phoX* and the relative proportion of genotypes in each clone library similar to *phoX* genes of different bacterial phyla. N-1, N-5, N-23, L-1, L-5, L-23, H-1, H-5, and H-23 represent the clone libraries constructed from the groups with varying densities of *Microcystis* biomass ranging from 15 to 1500 μg L^-1^ chlorophyll-*a* on day 1, 5, and 23, respectively; S represents the clone library constructed by the sedimentary sample on day 1.

The cluster analysis based on the UniFrac distances of bacterial *phoX* sequences among the clone libraries showed more obvious variations of genotypes in groups L and H than in group N during the decomposition of *Microcystis* blooms (Fig 4). The intergroup differences between groups L and H were more apparent than the within group differences on day 1 and 5. On day 23, the UniFrac distance between groups L and H was lower than distances earlier in the experiment. Along with decomposition, the differences between group N and the other two groups were diminishing, and the genotypes of groups L and H were more similar to group N on day 23.

According to the phylogenetic analysis result, the genotypes most similar to the *phoX* genes of *Betaproteobacteria* and *Cyanobacteria* always played a predominant role in group N during the experiment, whereas the L and H groups had a predominance of genotypes most similar to the *phoX* genes of *Alphaproteobacteria* on the day 1 and 5 of the experiment (Fig 4). The relative proportions of the alphaproteobacterial *phoX* in group H were higher than in group L on day 1 and 5. During day 1 and 5 in the L and H groups, the genotypes most similar to the *phoX* gene of *Microvirga* sp. WSM3557 played a dominant role, whereas the genotypes related to the *phoX* gene of *Synechococcus* sp. PCC 7002 dominated on the last day (day 23) of the experiment. In addition, the genotypes most similar to the *phoX* genes of *Alphaproteobacteria* and *Betaproteobacteria* also dominated in the surface sediments.

### CCA and abundance of bacterial *phoX* genotypes

**Fig 5:**
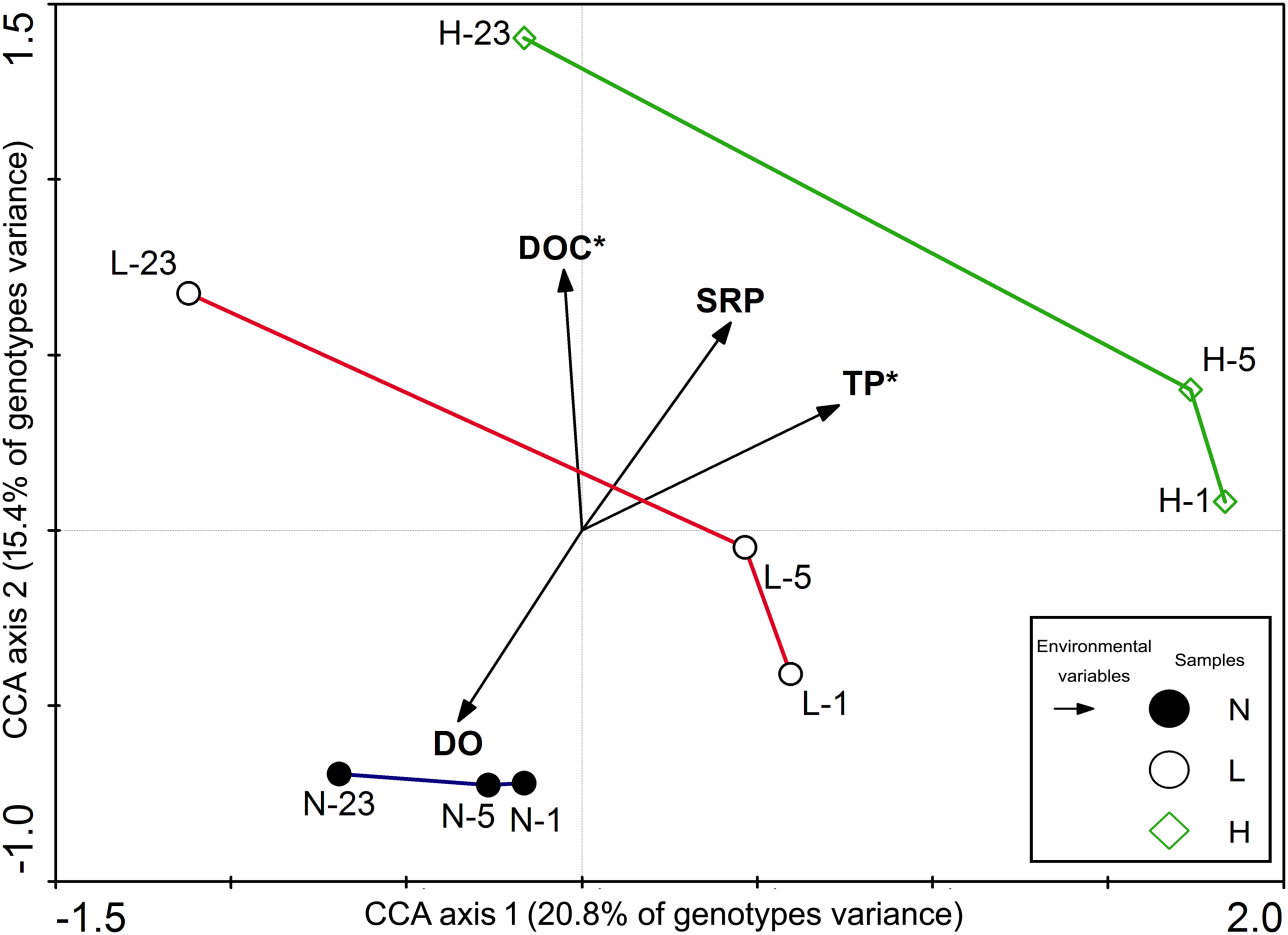
CCA biplot showing the variation of bacterial *phoX* genotypes in relation to environmental parameters. The significant physicochemical parameters are marked * (*P* < 0.05), and N-1, N-5, N-23, L-1, L-5, L-23, H-1, H-5, and H-23 represent the clone libraries constructed from the groups with varying densities of *Microcystis* biomass ranging from 15 to 1500 μg L^-1^ chlorophyll-*a* on day 1, 5, and 23, respectively; S represents the clone library constructed by the sedimentary sample on day 1. DO, dissolved oxygen; TP, total phosphorus; SRP, soluble reactive phosphorus; DOC, dissolved organic carbon.

The CCA of the variations of bacterial *phoX* genotypes and the physicochemical parameters of the water revealed that TP (*P* = 0.020, *F* = 2.79) and DOC (*P* = 0.048, *F* = 2.27) contents during the decomposition of *Microcystis* blooms were significant factors (Monte Carlo test), jointly contributing 36.2% of the variations in the bacterial *phoX* genes (Fig 5).

**Fig 6:**
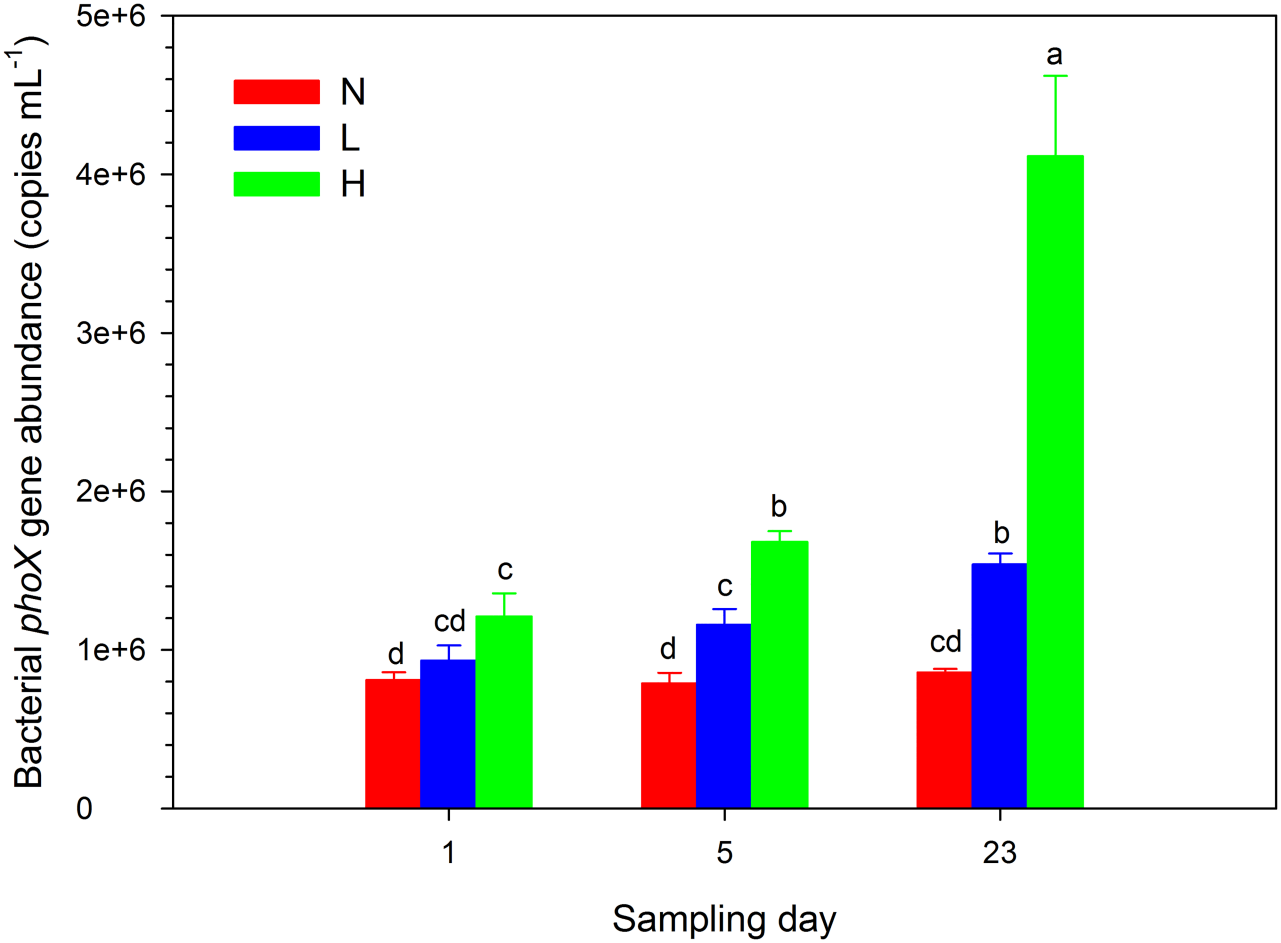
Bacterial *phoX* abundances of the three treatment groups on day 1, 5, and 23 of *Microcystis* blooms decomposition. N, L and H in the legend represent the three groups with ~15, ~150 and ~1500μg L^-1^ chlorophyll-*a*, respectively. The significance of difference represented by each letter shown in the figure. The different letter represents significant difference among groups (*P* < 0.05).

**Fig 7:**
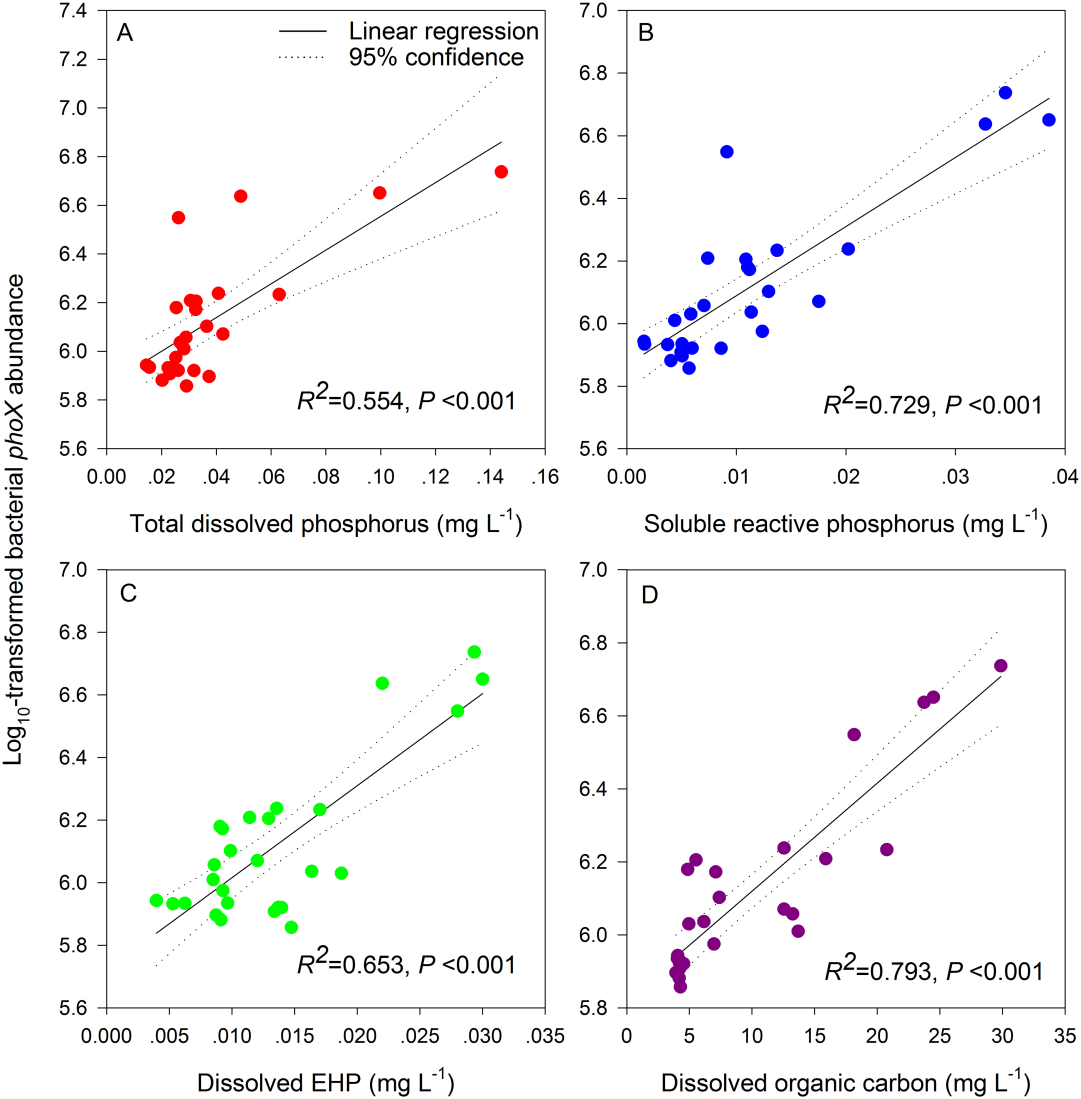
Correlations between the log-transformed bacterial *phoX* abundances and the corresponding average contents of total dissolved phosphorus (A), soluble reactive phosphorus (B), dissolved enzymatically hydrolysable phosphorus (C), and dissolved organic carbon (D) in water along with the decomposition of *Microcystis* blooms. The dots represent the 27 water samples from the three groups on days 1, 3, and 23.

The abundance of bacterial *phoX* genes in group N (7.88×10^5^ to 8.58×10^5^ copies mL^-1^) showed no significant variation throughout the experiment, whereas the bacterial *phoX* abundances in groups L and H gradually increased along with the decomposition of the *Microcystis* blooms (Fig 6). The abundance on the last day was higher than that on the first day, especially in group H, which showed an abundance of copy numbers reaching 4.11×10^6^ mL^-1^ (3.4 times that on the first day). In addition, the abundance of *phoX* in group H on the last day was remarkably higher than that in groups N and L. The contents of water DTP, SRP, dissolved EHP and DOC were significantly correlated with the values of the log_10_-transformed bacterial *phoX* abundances during the decomposition of the *Microcystis* blooms (Fig 7A–7D).

## Discussion

### Shifts in bacterial APases activity and *phoX* diversity

The decomposition of *Microcystis* blooms releases nutrients and organic matter that often cause an anaerobic hydro-environment [11], which was reflected by the decreased DO contents (S1 Fig) in this experiment. Before day 7 of the experiment, the concentrations of *Microcystis* aggregates were still high (Fig 2A). The *Microcystis* population might still need abundant SRP to sustain life, which explains the peak values of BAPA (Fig 3) and lowest values of SRP (Fig 2D) in all groups from day 5 to day 7. After day 7, massive decomposition of *Microcystis* blooms occurred, indicated by the significant decrease of Chl-*a* in both L and H groups (Fig 2A). Along with the massive release of SRP from the decomposed *Microcystis* aggregates, BAPA declined significantly after day 7 (Fig 3). Additionally, because the dead *Microcystis* aggregates were deposited in the surface sediments (S2 Fig), the TP contents that were contributed partly by the *Microcystis* aggregates decreased significantly in the water body (Fig 2B).

In this study, differences in bacterial *phoX* diversities were found among different groups on day 1 (Table 1), and an increasing gradient was observed in relative proportion of *phoX* genotypes similar to alphaproteobacterial *phoX* from groups N—H on day 1 (Fig 4). This phenomenon was attributed to the addition of different quantities of the *Microcystis* blooms into the three experimental groups. Previous investigations into bacterial community composition on the *Microcystis* aggregates in the algae-dominated regions of Lake Taihu in summer found a large number of *Alphaproteobacteria* populations attached on the aggregates [42]. Moreover, previous study also demonstrated that the *Alphaproteobacteria*, *Betaproteobacteria*, and unidentified bacteria dominated during the decomposition of *Microcystis* blooms in Lake Taihu [8, 11]. The addition of bacterial species, especially *Alphaproteobacteria*, increased the bacterial *phoX* diversity in each group of this experiment. In the previous study of Lake Taihu [25], the *phoX* genotypes similar to alphaproteobacterial *phoX* were always dominant in the algae-dominated regions during the summer and autumn.

Note that bacterial *phoX* diversity indexes (*S*_*Chao*_ and *H’*) in groups L and H decreased on day 5 and recovered on day 23 (Table 1). This phenomenon occurred because the genotypes similar to the *phoX* genes of *Betaproteobacteria* were not adaptable to an environment with massive accumulations of *Microcystis* blooms and explains why the genotypes similar to alphaproteobacterial *phoX* dominated during day 1 and 5 in this study (Fig 4). Although *Betaproteobacteria* was also the predominant bacterial population in eutrophic lakes [42–44], they prefer lakes with lower turbidity [45–46]. Previous investigation found more *phoX* genotypes of *Betaproteobacteria* in macrophyte-dominated lakes than in algae-dominated lakes [24].

In this study, several cyanobacterial genotypes were phylogenetically similar to those of *Synechococcus* sp. PCC 7002 (S3 Fig). *Synechococcus* is a type of pico-cyanobacteria widely found in oligotrophic and eutrophic lakes [47–48] and has a strong nutrient utilisation capability [49–50]. As autotrophic bacteria, their dependence on sunlight makes adapting to an environment with widely accumulated cyanobacterial bloom difficult [49]. Owing to the apparent decrease in water turbidity at the last stage of decomposition, the *Synechococcus* population, which requires sunlight, began to grow quickly. As a result, the proportion of genotypes similar to the cyanobacterial *phoX* increased to some extent in both L and H groups on the last day of the experiment.

Moreover, the clone library can only obtain the gene information of predominant species in the environment [51]. Because the overlap of bacterial *phoX* genotypes between sediments and water in the experiment was low (5.7%), the recovery of bacterial *phoX* diversities in all groups on day 23 should be related to the recovery of some subdominant species inhibited during the accumulation and decomposition of *Microcystis* blooms.

In addition, the *phoX* genotypes showed significant differences among the three groups on different days (Fig 4). CCA results showed that during the decomposition process the changes in the contents of TP and DOC were notably correlated with the variation of bacterial *phoX* genotypes (Fig 5). In Lake Taihu, 58% of TP content was attributable to EHP [16], an important source of bacterial nutrients that may influence bacterial communities via bottom-up regulation [52]. The DOC content, however, the carbon source needed for the growth and proliferation of bacteria, showed large differences at different stages of the decomposition process. Previous studies have shown that the community structure and composition of the bacteria shifted with increasing DOC content [53–54].

### Shifts in gene abundance along the decomposition of *Microcystis* blooms

During the decomposition process in the L and H groups, the abundance of *phoX* clearly increased, more significantly in the H group during the last stage of decomposition (Fig 6), which was attributable to two causes. First, with the decomposition of *Microcystis* blooms, a large amount of dissolved carbon, nitrogen, and phosphorus nutrients were released into the water, thus providing sufficient nutrition for the growth and proliferation of bacteria. The proliferation of bacteria at the end of decomposition has been verified in previous studies [8, 10]. Therefore, with the increasing number of bacteria, the number of extensively distributed APase-producing bacteria also grew, demonstrated by the significant correlations between the contents of TDP, SRP, dissolved EHP, DOC, and the log_10_-transformed bacterial *phoX* abundances during the experiment (Fig 7). Second, at the end of decomposition, the water turbidity decreased and the DO content increased. The improved water quality also allowed some recovery in bacterial diversity, thus favouring the growth of different populations. In addition, the abundance of *phoX* in the L and H groups during the early stage was higher than that in the N group, mainly because the *Microcystis* particles and the adherent heterotrophic bacteria in the fresh *Microcystis* aggregates added to the L and H groups contain more *phoX* genes.

In this study, however, we found that the peak values of bacterial APases activities and *phoX* gene abundances did not appear simultaneously in the same stage of *Microcystis* blooms decomposition (Figs 3 and 6). APases are always secreted by microbes when they face phosphorus limitation [20]. The decrease of SRP concentrations in groups L and H indicated that the densities of microorganisms utilized SRP and thus produced the high level of BAPA during days 5–7 (Fig 3). Moreover, although BAPA decreased during the last stage of this experiment, it was still the same as the initial level of the experiment in groups L and H (Fig 3). In other words, bacterial APases were still active in providing SRP for microorganisms after deep decomposition of *Microcystis* blooms.

As for PhoX-producing bacteria, the release of DOC and organophosphorus provided sufficient nutrients to sustain life. Previous study also demonstrated the increase of bacterial cells at the last stage of *Microcystis* blooms decomposition [8, 10]. In this experiment, the SRP content in the water at the end of decomposition accounted for <30% of the TDP content, indicating that most TDP was dissolved organic phosphate (DOP). Previous study has found that the return of rapid proliferation of *Microcystis* cells resulted in the rapid consumption of water SRP in spring [55]. The phytoplankton community was then limited by phosphorus nutrient availability and thus could exploit the large amount of DOP by extracellular APases in the water. This portion of the DOP therefore served as the main source of phosphorus for new outbreaks of *Microcystis* blooms [12]. In summer and autumn, the released organophosphorus and increased bacterial *phoX* abundance after decomposition of *Microcystis* aggregates provide sufficient nutrients and biological conditions for algal proliferation and are probably related to the regeneration of *Microcystis* blooms.

## Conclusions

1. The decomposition of *Microcystis* blooms released large amounts of SRP and dissolved organophosphorus (such as dissolved EHP and DOC) into the lake water, with the increasing gradient of released phosphorus contents from the low (~15 μg L^-1^) to high Chl-*a* density (~1500 μg L^-1^) group.
2. BAPA showed similar and significant variations during the decomposition of *Microcystis* blooms, with peak values during days 5~7 in different Chl-*a* density groups. Along with the decomposition process, BAPA in the three groups decreased dramatically and recovered to initial levels of the experiment at the last stage of decomposition.
3. The genotypes similar to the *phoX* gene of *Alphaproteobacteria* played a dominant role in the decomposition of *Microcystis* blooms with high (~1500 μg L^-1^) and moderate (~150 μg L^-1^) concentrations. At the end of decomposition, the proportion of genotypes similar to the *phoX* genes of *Betaproteobacteria* and *Cyanobacteria* increased, revealing the rapid response of bacterial *phoX* genotypes to the decomposition of *Microcystis* blooms.
4. At the end of *Microcystis* bloom decomposition, bacterial *phoX* abundances increased significantly in all the three experimental groups, which was attributed to the release of DOC and organophosphorus during the *Microcystis* blooms. The released organophosphorus and increased bacterial *phoX* abundance after decomposition of *Microcystis* aggregates could provide sufficient nutrients and biological conditions for the algal proliferation and are probably related to the regeneration of *Microcystis* blooms in eutrophic lakes.

## Supporting information

S1 Table Degenerate oligonucleotide primers used for amplification bacterial *phoX* gene fragment in this study. (DOC)

S1 Fig Variations in values of the physicochemical parameters in different experimental groups. N, L and H in the legend represent the three groups with ~15, ~150 and ~1500 μg L-1 chlorophyll-a, respectively.
(DOC)

S2 Fig Enrichment of sedimentary total phosphorus in different groups on day 23. N, L and H in the legend represent the three groups with ~15, ~150 and ~1500 μg L^-1^ chlorophyll-a, respectively. The different letter marked on each group represents a significant difference between two groups (one-way ANOVA, *P* < 0.05).
(DOC)

S3 Fig The maximum likelihood phylogenetic tree for bacterial *phoX* genotypes in different decomposition stages of the three experimental groups. The number of unique operational taxonomic units (OTUs) is in the first bracket and the number of sequences affiliated to the described OTUs is in the second bracket. This tree was calculated with 500 replicates of a bootstrap test based on the Jones-Taylor-Thornton model. The putative protein of *Stigmatella aurantiaca* DW4/3-1 (*Deltaproteobacteria*) *phoX* gene was selected as the output group. N-1, N-5, N-23, L-1, L-5, L-23, H-1, H-5, and H-23 represent the clone libraries constructed from the groups with varying densities of *Microcystis* biomass ranging from 15 to 1500 μg L^-1^ chlorophyll-a on day 1, 5, and 23, respectively.
(DOC)
