# Supplementary material for: Dynamics of phosphorus and bacterial phoX genes during the decomposition of Microcystis blooms in a mesocosm
Source: PLoS One. 2018 May 3;13(5):e0195205. doi: 10.1371/journal.pone.0195205 (PMC5933731; doi:10.1371/journal.pone.0195205)
Supplement: S1 Table — (DOC) [file pone.0195205.s001.doc]

**S1 Table. Degenerate oligonucleotide primers used for amplification bacterial *phoX* gene fragment in this study.** [1]

| **Primer name** | **Forward** | **Reverse** |
| --- | --- | --- |
| *phoX1* | 5’GARGARAAYTTYAACGGCTA | 5’GCCAKSACRWAVAGATCC |
| *phoX2* | 5’GARGAGAACWTCCACGGYTA | 5’GATCTCGATGATRTGRCCRAAG |
| *phoX3* | 5’GGGNACTTAYYTMACBTGYGAA | 5’GDCKATCCATBGKBGTTGC |

**References**

1. Sebastian M, Ammerman J. The alkaline phosphatase PhoX is more widely distributed in marine bacteria than the classical PhoA. ISME J. 2009; 3(5): 563-572.
