# Supplementary material for: Dynamics of phosphorus and bacterial phoX genes during the decomposition of Microcystis blooms in a mesocosm
Source: PLoS One. 2018 May 3;13(5):e0195205. doi: 10.1371/journal.pone.0195205 (PMC5933731; doi:10.1371/journal.pone.0195205)
Supplement: S1 Fig — N, L and H in the legend represent the three groups with ~15, ~150 and ~1500 μg L-1 chlorophyll-a, respectively. (DOC) [file pone.0195205.s002.doc]

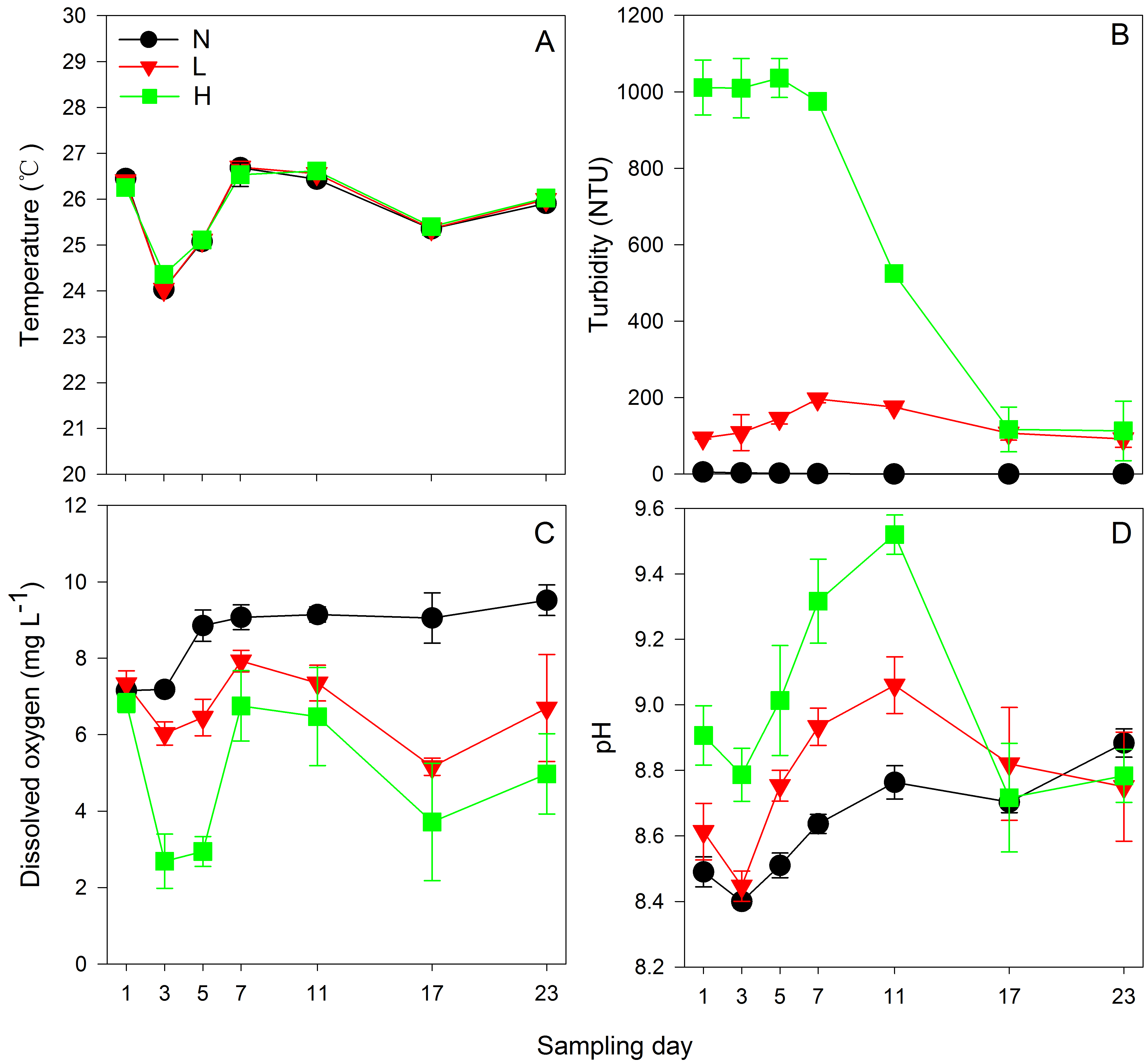


**S1 Fig. Variations in values of the physicochemical parameters in different experimental groups.**
