# Supplementary figures and images for: Dynamics of phosphorus and bacterial phoX genes during the decomposition of Microcystis blooms in a mesocosm
Source: PLoS One. 2018 May 3;13(5):e0195205. doi: 10.1371/journal.pone.0195205 (PMC5933731; doi:10.1371/journal.pone.0195205)

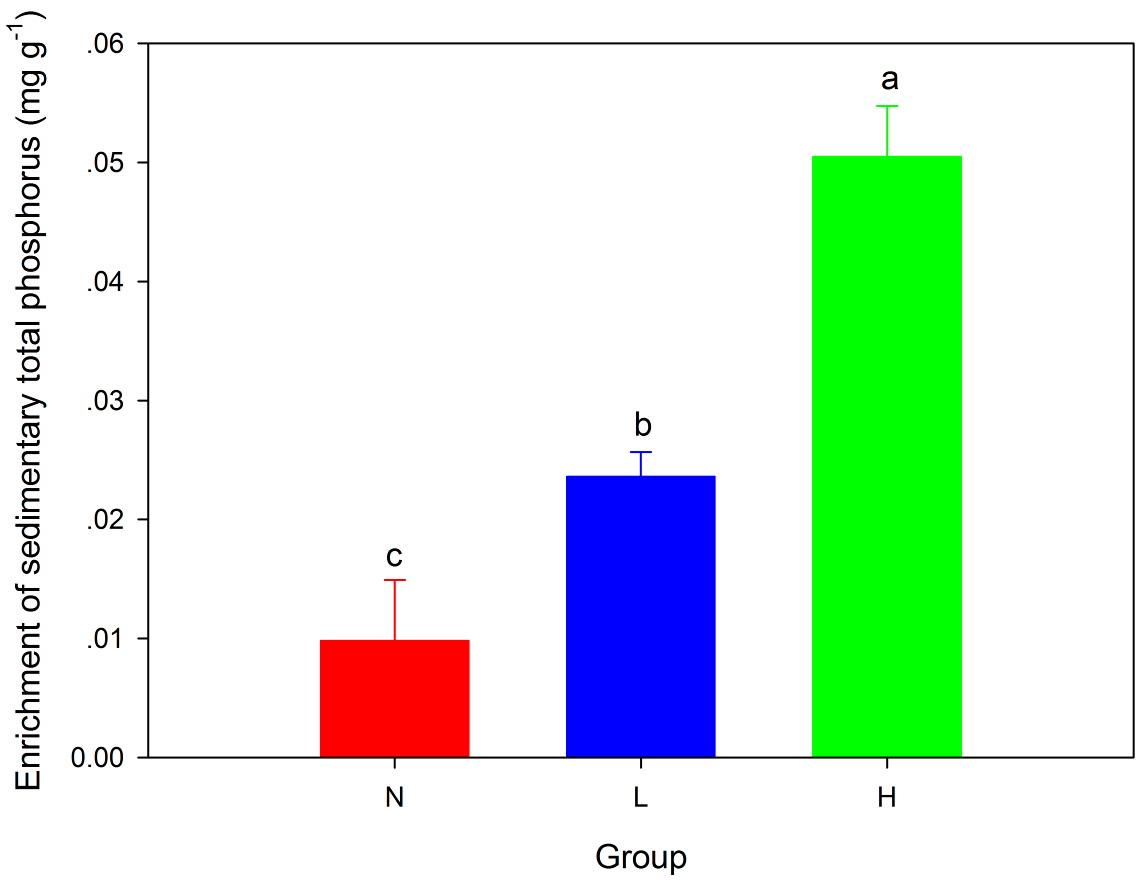


**S2 Fig. Enrichment of sedimentary total phosphorus in different groups on day 23.**

Supplement: S2 Fig — N, L and H in the legend represent the three groups with ~15, ~150 and ~1500 μg L-1 chlorophyll-a, respectively. The different letter marked on each group represents a significant difference between two groups (one-way ANOVA, P < 0.05). (DOC) [file pone.0195205.s003.doc]
