# Supplementary material for: Dynamics of phosphorus and bacterial phoX genes during the decomposition of Microcystis blooms in a mesocosm
Source: PLoS One. 2018 May 3;13(5):e0195205. doi: 10.1371/journal.pone.0195205 (PMC5933731; doi:10.1371/journal.pone.0195205)
Supplement: S3 Fig — The number of unique operational taxonomic units (OTUs) is in the first bracket and the number of sequences affiliated to the described OTUs is in the second bracket. This tree was calculated with 500 replicates of a bootstrap test based on the Jones-Taylor-Thornton model. The putative protein of Stigmatella aurantiaca DW4/3-1 (Deltaproteobacteria) phoX gene was selected as the output group. N-1, N-5, N-23, L-1, L-5, L-23, H-1, H-5, and H-23 represent the clone libraries constructed from the groups with varying densities of Microcystis biomass ranging from 15 to 1500 μg L-1 chlorophyll-a on day 1, 5, and 23, respectively. (DOC) [file pone.0195205.s004.doc]

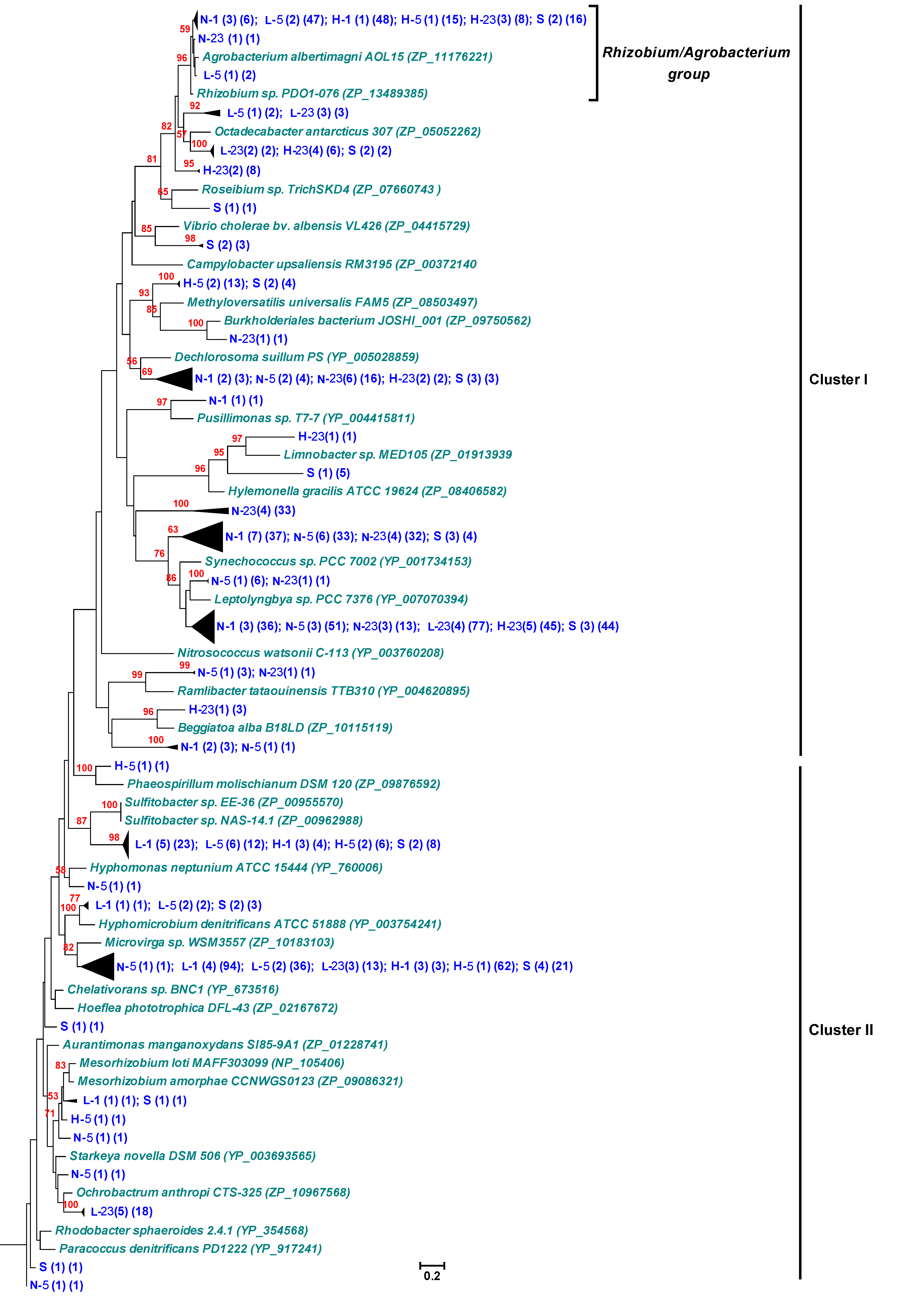


**S3 Fig. The maximum likelihood phylogenetic tree for bacterial *phoX* genotypes in different decomposition stages of the three experimental groups.**
